# Supplementary material for: Development of a computer-aided design software for the quantitative evaluation of aesthetic damage
Source: PLoS One. 2019 Dec 18;14(12):e0226322. doi: 10.1371/journal.pone.0226322 (PMC6919621; doi:10.1371/journal.pone.0226322)
Supplement: S1 Code site — 1. (ZIP) [file pone.0226322.s001.zip › [EESC_jr][Projeto]Relatorio_07_21/reports/templates_zip/gentelella-master/vendors/Flot/examples/resize/index.html]

Flot Examples: Resizing


## Resizing

Sometimes it makes more sense to just let the plot take up the available space. In that case, we need to redraw the plot each time the placeholder changes its size. If you include the resize plugin, this is handled automatically.

Drag the bottom and right sides of the plot to resize it.

Copyright © 2007 - 2014 IOLA and Ole Laursen
